# Signal Peptidase Complex Subunit 1 and Hydroxyacyl-CoA Dehydrogenase Beta Subunit Are Suitable Reference Genes in Human Lungs

**DOI:** 10.5402/2012/790452

**Published:** 2011-12-28

**Authors:** Issac H. K. Too, Maurice H. T. Ling

**Affiliations:** ^1^Department of Biological Sciences, National University of Singapore, Singapore 117543; ^2^Department of Zoology, The University of Melbourne, Parkville, Victoria 3010, Australia

## Abstract

Lung cancer is a common cancer, and expression profiling can provide an accurate indication to advance the medical intervention. However, this requires the availability of stably expressed genes as reference. Recent studies had shown that genes that are stably expressed in a tissue may not be stably expressed in other tissues suggesting the need to identify stably expressed genes in each tissue for use as reference genes. DNA microarray analysis has been used to identify those reference genes with low fluctuation. Fourteen datasets with different lung conditions were employed in our study. Coefficient of variance, followed by NormFinder, was used to identify stably expressed genes. Our results showed that classical reference genes such as GAPDH and HPRT1 were highly variable; thus, they are unsuitable as reference genes. Signal peptidase complex subunit 1 (SPCS1) and hydroxyacyl-CoA dehydrogenase beta subunit (HADHB), which are involved in fundamental biochemical processes, demonstrated high expression stability suggesting their suitability in human lung cell profiling.

## 1. Introduction

According to American Cancer Society, lung cancer is estimated to account for 27.6% of all cancer-related deaths in America in 2010. In a report surveying cancer occurrence in Singapore from 1968 to 2007 published by National Cancer Centre Singapore, lung cancer is rated as the second and third highest occurrence of cancer in Singaporean men and women, respectively, (http://www.nccs.com.sg/pat/file/Report_1968_2007.pdf). Although lung cancer is preventable at early stage, it is usually diagnosed at advanced stage of disease, which is usually too late for current medical intervention and subsequently causes mortality [1]. Therefore, there is a need to profile gene expressions of lung epithelial cells to advance current treatment modalities.

Quantification of gene expressions allows for the analysis of different genes threshold regulation [2]. Profiling of gene expression by the mean of quantitative real-time polymerase chain reaction (qRT-PCR), Northern blot, and DNA microarray analysis [3] allow the study of tumors-related biomarkers regulation and the prognosis of disease stage [4] for lung cancer patient [5]. However, a number of variables, such as selected cell types, mRNA extraction and handling techniques, and analytical quantification approaches [6] may result in different gene expression measurements and affect analysis accuracy [2]. In order to address these variations, normalization that usually involves a group of calibrating genes is employed in many gene expression quantification studies [2]. 

It has been suggested that calibrating genes are generally used as housekeeping gene in gene expression studies [2] due to their highly stable expression [7]. The ideal set of housekeeping genes should express stably and consistently across all samples [8]. To date, the selection of reference genes can be achieved by few mathematical method, such as geNorm [9], BestKeeper [10], and NormFinder [11] to evaluate gene expression stability in qRT-PCR [2]. BestKeeper [10] and geNorm [9] were based on pairwise correlation and had been shown to be sensitive to coregulated genes [12]. On the other hand, NormFinder is less sensitive to coregulated genes as it took account of the variations across subgroups [11]. However, these tools are usually applicable in a small sample size cDNA panel to determine the most stable reference genes [13]. Microarray is commonly used in quantification of gene expression with the advantage of speed and high throughput [14, 15]. Due to the ability to analyze the expression of thousands of genes in an experiment, microarray presents a suitable resource for the analysis and identification of reference genes [16]. Previous studies [17, 18] had shown that the analysis of coefficient of variance (CV) values of each gene is able to determine the stably expressed genes which can be used in gene expression quantification studies. 

The recent lung cell lines profiling studies have widely employed a group of common endogenous control genes, such as GAPDH and beta-actin [1] to measure gene expression changes. However, different studies had shown the variability of GAPDH expression in non-small cell lung cancer [19, 20], suggesting that GAPDH may not be the most suitable housekeeping gene to investigate gene expression of lung cells. In addition, some studies [1, 2, 21, 22] had identified that the mRNA expression levels of different housekeeping genes such as GAPDH and beta-actin may vary under different conditions [2]. This suggests that no single housekeeping gene is universal in all types of gene expression studies [1]. Previous studies had shown that ACTB and SDHA were effectively used as reference genes in breast tumour studies [23] while IPO8 and MARK3 were stably expressed in human lung specimens [1] and mouse liver [17], respectively. Besides, mouse liver and mouse adrenal gland study also showed that EIF2A and PPIB were stably expressed [24]. Other studies also indicated that EEF1A1 expressed consistently in human cervical tissues [25] while B2M and RPL29 were well used as reference genes in human stomach tissue studies [26]. High expression stability showed that RPL32, GAPDH, POLR2A, TBP, PGK1, and RPL4 were demonstrated as the most reference genes in rodent and human heart gene expression studies [27] while RPS18 was stably expressed in head and neck squamous cell carcinoma [21]. In addition, GUSB was generally used as reference gene in human ovarian studies [28] while RPS4X and RPL13A were useful in quantifying the gene expression in larvae of flatfish [29] and osteoarthritic canine articular tissues [2], respectively. This proposes that different housekeeping genes may express inconsistently in different species or organs. 

In this study, we investigated a group of endogenous genes in human lung and analyzed their CV values to suggest their suitability and stability as reference genes for future quantitative gene expression studies. Our results suggest that SPCS1 and HADHB are more stably expressed in human lungs than any of the 20 reference genes found by previous studies.

## 2. Materials and Methods

### 2.1. Microarray Data

Fourteen microarray data sets studying human lung epithelial from Gene Expression Omnibus (GEO, http://www.ncbi.nlm.nih.gov/geo/) were used. All of which employed Affymetrix Human Genome U133 Plus 2.0 Array (GPL570) containing 54,676 probes. This allows for comparisons across different data sets. The fourteen datasets were: bronchial epithelial cells at 4 and 24 hours following treatment with respiratory syncytial virus (GDS2023), airway epithelial cells of phenotypically normal smokers (GDS2486 and GDS2491), airway epithelia from healthy individuals 7 and 14 days following injury by epithelial denudation (GDS2495), epithelial and mesothelial lung cell lines at various time points up to 7 days after exposure to asbestos (GDS2604), 28 days air-liquid cultured airway epithelial cells (GDS2615), analysis of resveratrol treated lung carcinoma A549 cells (GDS2966), analysis of house dust mite (HDM) extract-exposed H292 bronchial epithelial cells (GDS3003), bronchial epithelial cells exposed to cigarette smoke from a typical full flavor brand for up to 24 hours (GDS3493), bronchial epithelial cells exposed to cigarette smoke from a typical light flavor brand for up to 24 hours with (GDS3494), lung adenocarcinoma CL1-5 cells overexpressing Claudin-1 (CLDN1) (GDS3510), comparison of non-small cell lung cancer histological subtypes: adenocarcinomas (AC) and squamous cell carcinomas (SCC) (GDS3627), analysis of normal lung WI-38 fibroblasts exposed to various concentrations of the carcinogen benzo[a]pyrene-diol epoxide (BPDE) (GDS3706), and analysis of A549 epithelial cells treated for up to 72 hours with TGF-beta (GDS3710). 

### 2.2. Z-Score Normalization across Data Sets

Individual GEO datasets were normalized by arithmetic mean transformation and Z transformation to construct parallel and comparable datasets based on the method described in [30]. Within a microarray data, specific gene with multiple probes may show different intensities. In each dataset, the intensity value for each probe (Probe_Initial_) was used to compute the average probe intensity for each dataset (*μ*_InitialProbe_). Next, by assuming the standard arithmetic mean of all the probes as 1000 for the overall combined microarray datasets (*μ*_Assumed_), the correction factor for the average probe intensity for each datasets is constructed as a quotient of *μ*_Assumed_ and *μ*_InitialProbe_; Correction Factor = *μ*_Assumed_/*μ*_InitialProbe_. The transformed intensity of each original probe (Probe_Transformed_) is then calculated using the equation, Probe_Transformed_ = Probe_Initial_ × Correction Factor. The Probe_Transformed_ was then used to calculate Z-score by the equation, Z_score_ = (Probe_Transformed_ − *μ*_Assumed_)/SD_Dataset_, where SD_Dataset_ is the standard deviation of the original dataset from which the probe (Probe_Initial_) originates. The Z-scores for each probe across different original datasets will then be comparable.

### 2.3. Ranking of Reference Genes 

Arithmetic mean and standard deviation of the Z-scores were derived for all the genes in the fourteen datasets. The coefficient of variance (CV) values were defined as the quotient of standard deviation to the arithmetic mean [17]. By constructing the CV values for all the probes of the chosen reference genes from the fourteen datasets, the mean CV values for each reference gene were generated. Using the Z-transformed dataset, 10% of the probes with the lowest CV were isolated [17] and filtered for any duplicate probes with CV that is more than 10% of lowest CV. In this case, the CV will be the quotient of standard deviation of the Z-score to the arithmetic mean of the Z-score. For example, if gene A is represented in the microarray as 3 probes and all 3 probes are found in list of 10% lowest CVs, gene A is retained. On the other hand, if gene B is represented in the microarray as 3 probes and one of the probe is not in the list of 10% lowest CV, gene B is removed from the list. Only probes with gene symbols were included in the list. The filtered list was then analyzed with NormFinder [11] using the normalized microarray data as expression values. Due to the reason that NormFinder has the limitation of analyzing zero and negative values, the normalized expression values of the fourteen datasets which were lower than 0.0001 or close to zero value were transformed into the absolute values or 0.0001, respectively. 

### 2.4. Comparing NormFinder and CV 

Spearman's correlation was used to determine the correlation between stability index generated by NormFinder and CV values using the equation, *r* = 1 − [(6∑*d* ^2^)/*n*(*n* ^2^ − 1)] where *r* is the Spearman's correlation, *d* is the difference in the rank of two parameters, and *n* is the sample size. The *t*-statistic was calculated by *r*[(*n*−2)/(1−*r* ^2^)]^1/2^, which was used to test for the null hypothesis of no correlation with (*n* − 2) degrees of freedom.

## 3. Results

Twenty housekeeping genes from previous studies [1, 2, 17, 19–21, 23–28] were analyzed and their mean of mean CV values was tabulated in Table 1. Our results showed that different housekeeping genes have indicated diverse gene expression fluctuations within the human lung tissues with the CV values ranging from 0.42 to 2.38. Within these housekeeping genes, Eukaryotic translation initiation factor 2A (EIF2A) showed the least variation among the 20 housekeeping genes with the CV value of 0.4225 followed by Peptidyl-prolyl *cis*-trans isomerase B (PPIB, 0.4394), 40S ribosomal protein S4 (RPS4X, 0.4455), 60S ribosomal protein L29 (RPL29, 0.4498), and Ribosomal protein L4 (RPL4, 0.4623) whilst Phosphoglycerate kinase 1 (PGK1) showed the highest fluctuation with the CV value of 2.3760. The results showed that Microtubule affinity-regulating kinase 3 (MARK3), TATA-binding protein (TBP), and PGK1 have the great fluctuations in their gene expression level with the mean CV values of 1.6441, 2.0988, and 2.3760, respectively. Based on the mean CV values, the gene expressions of MARK3 fluctuated at least two times more compared to EIF2A, while the fluctuation magnitude in the gene expression level of TBP and PGK1 corresponding to EIF2A was about four times larger. 

By extracting the 10% probes with the lowest CV values from the transformed Z-score data, 5,458 probes were extracted from the original 54,676 probes. After removing those gene probes without specific gene name, the total probes number left 2,213 probes. Among these 2,213 probes, specific genes that only fall within the lowest 10% CV but not vice versa were found to be 743 genes, which are originally encoded by 932 probes. The analysis result showed that among the 20 housekeeping genes only EIF2A, PPIB, RPL4, 60S ribosomal protein L13a (RPL13A), 60S ribosomal protein L32 (RPL32), and 40S ribosomal protein S4 (RPS4X) were found in the lowest 10% CV subset. NormFinder [11] was used to analyze these 932 probes, and their stability indices were tabulated in Table 2. Our analysis showed that signal peptidase complex subunit 1 (SPCS1) and hydroxyacyl-CoA dehydrogenase/3-ketoacyl-CoA thiolase/enoyl-CoA hydratase, beta subunit (HADHB), have the highest stability of rank 1 and 2 with the stability index of 0.326 and 0.355, respectively, followed by 71 genes with the stability index of 0.360. EIF2A, PPIB, RPL4, RPL13A, RPL32, and RPS4X have the rank ranging from 305 to 730. The mean Z-score of the probes within the lowest 10% CV ranges from −0.4073 to 11.7692. The mean Z-scores of HADHB and SPCS1 are 1.8419 (61.8 percentile) and 2.8516 (75.8 percentile), respectively.

The standard deviation of CV values and NormFinder stability index were 0.077 and 0.265, respectively. Due to a more than 3 times difference in standard deviation, homoscedasticity (constant variance) was not assumed. Thus, Spearman's rank correlation coefficient was carried out to determine the correlation of stability index by NormFinder and CV values which showed that the sum of *d* ^2^ is 85109486 (Spearman's rank correlation coefficient = 0.369) and the *P* value was 1.79 × 10^−31^. Since the *P* value was lesser than 0.01, the null hypothesis is rejected, indicating that there is correlation between the stability index from NormFinder and CV values.

## 4. Discussion

The accuracy of human lung specimens profiling mainly relied on the gene expression study of the lung epithelial cells using the reference genes that must remain stable and constant [8]. Therefore, the selection criteria to identify suitable reference genes must be stringent such that identified genes can be globally used in human lung profiling [1]. Previous studies [1, 2, 21, 22] had shown that not all commonly used housekeeping genes such as GAPDH and beta-actin can be utilized in different clinical samples as those gene expression levels may vary under different conditions [2]. In addition, some studies [19, 20] also demonstrated that the GAPDH is not the desired housekeeping gene in human lung gene expression study. Hence, this study employs a pool of microarray datasets to identify genes with low expression variation that can be used in human lung gene profiling.

The determination of expression stability in our study is dependent on two parameters, CV values and NormFinder Stability Index [11]. NormFinder was used in this study as it is less sensitive to coregulated gene expressions compared to geNorm and BestKeeper [12]. In our study, CV values were generated based on the probe intensity deviation of the microarray data. Low CV values indicate that those probes on the microarray chip that encode for the same gene have low fluctuation in gene expression. Similarly, low NormFinder Stability Index [11] indicates low fluctuation in gene expression. 

In this study, CV serves as a basic filter for genes with low expression variation. This is useful when the sample size is large as the performance of CV is linear to sample size. However, CV does not take into account systematic variations such as those introduced by inaccuracy in sample preparation. Thus, CV can only be used as a categorical filter of gene expression into a striation of classes. NormFinder is employed as the fine diagnostic tool to further determine the stability of gene expression within the class of genes with low expression variation to identify the most stably expressed gene. 

However, it is plausible that the results of CV and NormFinder analysis may be correlated as both methods had been used to identify reference genes within a dataset [2, 17]. Our results suggest that CV and NormFinder stability are significantly correlated to each other (*P* value = 1.79 × 10^−31^). However, the strength of this correlation is difficult to establish as the significance in *P* value did not indicate the correlation strength. At the same time, the comparison between CV, NormFinder [11], geNorm [9], and BestKeeper [10] requires a more comprehensive study using a datasets spanning across different tissues and organisms.

Our results showed that common housekeeping genes such as GAPDH and HPRT1 that had been used widely in many gene expression studies are not the appropriate reference genes to be employed in the human lung gene expression profiling. The mean CV values of GAPDH and HPRT1 were found to be higher than EIF2A which indicated that GAPDH and HPRT1 did not express stably compared to EIF2A. Although GAPDH and HPRT1 had been used as general reference genes in most gene expression studies [31, 32], our results suggested that the expression of GAPDH and HPRT1 in human lung epithelial cells varies significantly as they are not within the lowest 10% CV, suggesting that they are not suitable reference genes for human lung epithelial cells.

GADPH has a role in glycolysis pathway, DNA replication and repair, and RNA transportation [33] while HPRT1 is involved in magnesium ion binding [34], purine salvage pathway [35], and protein binding [36] in human body. Since both genes function as the building block of fundamental biochemical pathway in human body, they are assumed to be expressed stably most of the time under normal condition. Therefore, many studies have used GAPDH [1, 2] and HPRT1 [37] as the reference gene. Nevertheless, the lung specimens of these 14 datasets have been treated in different conditions. These conditions may upregulate or downregulate certain gene functions and in turn affect the gene product. In fact, one reference gene may be sufficient to profile a specific specimen, yet the stability may not hold true for other specimen or organisms [2]. As the result, the expression level of GAPDH and HPRT1 varied and affect their suitability as reference gene in human lung epithelial cell lines profiling.

Among the twenty selected housekeeping genes, only EIF2A, PPIB, RPL4, RPL13A, RPL32, and RPS4X were found within the list of lowest 10% CV. However, their stability indices were significantly higher compared to SPCS1 and HADHB which showed the stability index of 0.326 and 0.355, respectively, followed by 71 genes with the stability index of 0.360, suggesting that the expressions of SPCS1 and HADHB are considerably stable among the datasets analyzed. This suggests that both SPCS1 and HADHB are suitable reference genes in the human lung studies. Our results indicated that the mean Z-scores of HADHB and SPCS1 are 61.8 percentile and 75.8 percentile, respectively, suggesting that HADHB and SPCS1 are suitable reference genes for genes with average to high expression. Further studies are needed to determine their validity for genes with low expression.

The datasets used in this study include lung cell lines (GDS2604), bronchial epithelial cells (GDS2486 and GDS2491), A549 lung adenocarcinoma cells line (GDS2966), lung adenocarcinoma CL1-5 cells line (GDS3510), and squamous cell carcinomas (GDS3627). This suggests that SPCS1 and HADHB are suitable reference genes for primary lung tissues, lung cell lines, lung tumours, and bronchial epithelial cells. In addition, the datasets used in this study include healthy bronchial tissues and bronchial tissues from smokers (GDS2486), as well as normal lung tissues (GDS2615). This further suggests that SPCS1 and HADHB are suitable reference genes for both healthy and perturbed tissues.

SPCS1 is the gene located at 3p21.1 that functions as signal peptidase complex in the signal sequences cleavage of most secretory and membrane proteins [38]. Most secretory proteins are required to be translocated into endoplasmic reticulum (ER) membrane in order to allow the proteins to fold and assemble in a proper way before they are transported to the Golgi apparatus. Proteins that fail to assemble or fold into their native state will be translocated back across ER membrane to cytoplasm and undergo degradation [39]. In order for this to take place, the signal sequences on the protein must be cleaved during protein synthesis [38]. SPCS1 plays a role in cleavage of signal sequences of most secretory proteins to enable their translocation across the ER membrane [38]. 

HADHB is located at 2p23 and is involved in mitochondrial betaoxidation of long-chain fatty acids [40]. Previous study [41] had shown that different organs have different tendencies in fatty acid distribution, as liver is served as the largest reservoir for fatty acids followed by brain and lung. Since lung is one of the reservoirs for fatty acid, HADHB, a lipase, will be likely expressed more stably in the lung in order to degrade fatty acids and generate ATP. 

However, these two genes had not been used as reference genes in gene quantification studies.

A previous study showed that Importin 8 (IPO8) has expressed stably in human lung clinicopathological specimens [1]. However, its mean CV value of 0.8735 (Table 1) indicated that IPO8 has high gene expression fluctuation across different human lung specimens. Besides, the mean CV value of IPO8 was absent from the lowest 10% CV values subset that indicates that IPO8 may not be a good reference gene in human lung gene profiling as compared to SPCS1 and HADHB.

The identification of reference genes for the tissue of study is pivotal in analyzing the results of gene expressions [2]. Using an expressionally variant gene as reference gene in expression studies is likely to result in erroneous interpretation [1]. Hence, it begs the question of whether there is an organ-specific reference gene across a taxonomical lineage of organisms or an organism-specific reference gene, or even more globally, whether there is a universal reference gene within a taxonomical hierarchy such as a family-specific reference gene or an order-specific reference gene. The suggestion of a possible universal reference gene within a taxonomical hierarchy does imply the presence of organism-specific reference gene. 

A previous study [27] had suggested the low availability of organ-specific reference gene across different organisms, showing that RPL4 was found to be the most suitable reference gene in mouse myocardium but not in human heart. Although this suggests against having a universal housekeeping gene across different organism in gene profiling studies [8], it may be possible that there can be common reference genes for specific organs across a few organisms. However, this preposition requires an extensive study to identify genes across a wide variety of organisms and organs. In this study, SPCS1 and HADHB were identified as the most suitable reference genes in human lung profiling. As SPCS1 and HADHB had not been used as reference genes, there is no evidence to suggest that these genes are stably expressed in other organs even though they may be suitable reference gene candidates for lung profiling in other organisms but this requires further studies. In addition, another study [42] also suggested the absence of robust reference gene which is organism specific by demonstrating that GAPDH is not expressed consistently across the human subject candidates and resulted in high fluctuation of RNA expression. Taken together, this suggests the need to identify suitable housekeeping gene for every organ of every organism, by normalizing the gene expression using stringent mathematical model. 

## Figures and Tables

**Table 1:** Twenty housekeeping genes, together with *SPCS1* and *HADHB*, and their mean CV values across fourteen datasets. Within a microarray, multiple probes encode for a specific gene. By computing the CV value of one probe of a specific gene across the fourteen datasets, the mean CV values of all the probes that encode for the same gene were then calculated. *SPCS1* and *HADHB* are stably expressed genes found in this study and are added to the list for comparison.

Gene symbols	Mean of CV	Gene symbols	Mean of CV
*SPCS1*	0.2279	*GAPDH*	0.5708
*HADHB*	0.3363	*HPRT1*	0.6222
*EIF2A*	0.4225	*SDHA*	0.6563
*PPIB*	0.4394	*B2M*	0.6824
*RPS4X*	0.4455	*EEF1A1*	0.8094
*RPL29*	0.4498	*POLR2A*	0.8569
*RPL4*	0.4623	*IPO8*	0.8735
*RPL32*	0.4669	*GUSB*	0.9005
*RPL13A*	0.4725	*MARK3*	1.6441
*RPS18*	0.4962	*TBP*	2.0988
*ACTB*	0.5335	*PGK1*	2.376

**Table 2:** Stability of housekeeping genes generated by NormFinder [11]. By extracting the pool of Z-transformed probes intensities with the lowest 10% CV, the remaining 5,458 probes (out of 54,676 probes) were further selected to eliminate undefined genes. The selected 743 genes was input in NormFinder [11] to generate the stability value for each gene as a direct measure for the estimated expression variation and rank them accordingly. *SPCS1* and *HADHB*, which were found to have the lowest CV values, demonstrated the lowest stability index and ranked as first and second, respectively.

Gene symbols	NormFinder stability index	Rank
*SPCS1*	0.326	1
*HADHB*	0.355	2
*EIF2A*	0.691	371
*PPIB*	0.862	630
*PPIB*	0.867	634
*RPL13A*	0.651	305
*RPL13A*	0.811	548
*RPL13A*	0.832	587
*RPL13A*	0.911	689
*RPL13A*	0.950	730
*RPL32*	0.739	454
*RPL4*	0.723	428
*RPL4*	0.789	518
*RPL4*	0.805	541
*RPS4X*	0.777	499
*RPS4X*	0.838	592
